# Surgical management of first‐time patellar dislocations in paediatric patients may lower rates of redislocation compared to conservative management: A systematic review and meta‐analysis

**DOI:** 10.1002/ksa.12524

**Published:** 2024-10-30

**Authors:** Benjamin Blackman, Joshua Dworsky‐Fried, Dan Cohen, David Slawaska‐Eng, Lauren Gyemi, Nicole Simunovic, Devin Peterson, Olufemi R. Ayeni, Darren de SA

**Affiliations:** ^1^ School of Medicine University of Limerick Limerick Ireland; ^2^ Michael G. DeGroote School of Medicine McMaster University Hamilton Ontario Canada; ^3^ Division of Orthopedic Surgery, Department of Surgery McMaster University Hamilton Ontario Canada

**Keywords:** dislocation, management, MPFL, paediatric, patella

## Abstract

**Purpose:**

The purpose of this study is to assess whether early surgical intervention for first‐time patellar dislocations in paediatric patients is superior to conservative management. We hypothesized that surgical intervention would lead to lower redislocation rates compared to conservative treatment.

**Methods:**

Three online databases (PubMed, MEDLINE and EMBASE) were searched from inception to 14 March 2024 to identify studies investigating the management options for acute first‐time patellar dislocations in paediatric patients. Data pertaining to patient demographics, patient management, redislocation rates and Kujala scores, evaluating function, were abstracted. Weighted means and meta‐analyses were conducted to compare rates of redislocation, as well as Kujala scores. The quality of included studies was assessed using the methodological index for non‐randomized studies criteria for non‐randomized studies and the ROB2 tool for randomized controlled trials (RCTs).

**Results:**

A total of 11 studies and 761 patients were included in this review. The weighted mean post‐operative combined rates of redislocation in the surgical group was 25.1%, compared to 46.4% in the conservative group at a mean follow‐up of 53.2 months (12–168). The relative risk (RR) of redislocation was 0.82 (95% confidence interval [CI]: 0.65‐1.04, *I* ^2^ = 0%, *p* = 0.11), favouring surgery compared to conservative management. A subgroup meta‐analysis of two recent RCTs with 110 patients demonstrated an RR of redislocation of 0.53 (95% CI: 0.31–0.91, *I* ^2^ = 0%, *p* = 0.02), favouring surgery. Kujala scores among three comparative studies showed a mean difference of −2.7 (95% CI: −6.1 to 0.68, *I* ^2^ = 0%, *p* = 0.12), favouring conservative treatment. The weighted mean redislocation rate in 131 patients undergoing medial patellofemoral ligament reconstruction (MPFLR) was 3.1%, compared to 39.4% in 203 patients undergoing other surgical procedures, such as lateral release and medial imbrication, Roux‐Goldwaith and MPFL repair. Furthermore, the conservative groups experienced a complication rate of 0.9% compared to 2.9% across the surgical groups.

**Conclusion:**

Surgical management for first‐time patellar dislocations in a paediatric population, particularly MPFLR, may be more beneficial in lowering redislocation rates than conservative management. No significant differences in Kujala scores were found.

**Level of Evidence:**

Level IV.

**Abbreviations**
MINORS: methodological index for non‐randomized studies
MPFL: medial patellofemoral ligament
MPFLR: medial patellofemoral ligament reconstruction
MTR: medial tissue repair
PRISMA: preferred reporting items for systematic reviews and meta‐analyses
RCT: randomized controlled trial
ROB2: risk‐of‐bias tool for randomized trials
RTS: return to sports
TTO: tibial tubercle osteotomy

## INTRODUCTION

Patellar dislocations comprise roughly 3% of knee injuries, with most occurring in younger and active patients [9, 18]. The prevalence of patellar dislocations in patients under 18 is estimated to be around 29 per 100,000, compared to 5.8 in 100,000 in their adult counterparts [9, 14].

First‐time dislocations are often managed conservatively; however, some studies cite recurrent instability rates following conservative treatment from 35% to 71% [3, 14, 33]. Patellar dislocations can also be managed surgically using techniques, such as MPFL repair [17], MPFL reconstruction (MPFLR) [29], tibial tubercle osteotomy (TTO) [32], trochleoplasty [8], Roux‐Goldwaith procedure [42] and lateral retinacular release (LRR) and medial imbrication [28, 39]. Surgical modalities are often reserved for patients with recurrent instability, anatomical risk factors such as trochlear dysplasia, or when there are concomitant osteochondral injuries requiring fixation [6]. Nevertheless, the evidence behind an initial conservative treatment remains limited. Two recent reviews on acute first‐time patellar dislocations in adults found that both MPFL reconstruction and repair resulted in lower redislocation rates [5, 23], indicating that surgery may play a role in adult first‐time dislocations.

The ideal management of patellar dislocations in paediatrics is more complex, as the skeletal maturity level can influence the likelihood of recurrent instability [11]. Paediatric patients tend to have higher recurrence rates and associated complications following patellar dislocation [43]. Additionally, recurrent patellar dislocation puts patients at increased risk of developing patellofemoral arthritis, highlighting the importance of preventative measures in maintaining joint health [37]. However, little consensus remains on the optimal management of first‐time patellar dislocations in this higher‐risk population [2]. This review aims to determine the effect of early surgical treatment versus conservative measures in managing first‐time acute patellar dislocations in a paediatric population. We hypothesized that patients undergoing early surgical intervention after first‐time patellar dislocation would demonstrate lower redislocation rates than those undergoing conservative treatment.

## METHODS

The preferred reporting items for systematic reviews and meta‐analyses (PRISMA) guidelines for coordinating and reporting systematic reviews were followed during the development of this research [21, 26].

### Search criteria

Three online databases (PubMed, MEDLINE and EMBASE) were searched from database inception to 14 March 2024 to identify studies that investigated the management options for acute first‐time patellar dislocations in paediatric patients. Comprehensive search terms, including ‘MPFL’, ‘medial patellofemoral ligament’, ‘patellar instability’, ‘patellar dislocation’, ‘repair’, ‘reconstruction’, ‘treatment’, ‘rehabilitation’, ‘rehab’, ‘conservative treatment or conservative’, ‘adolescent’, ‘paediatrics’ and ‘skeletally immature’, were used (Appendix S1).

The research question and study eligibility were determined a priori. Studies were selected for inclusion if they met the following criteria: (1) surgical treatment of first‐time patellar dislocations, (2) patients under the age of 18, (3) level of evidence I–IV, (4) clinical and/or functional outcomes reported, (4) human studies and (5) studies published in the English language. Exclusion criteria included (1) history of >1 patellar dislocation or recurrent dislocations, (2) adult patients, (3) textbook chapters, (4) conference abstracts, (5) biomechanical or cadaveric/animal studies, and (6) case studies and case series with five or fewer patients. References of included studies and pertinent review papers were manually searched to ensure all means of study identification were exhausted.

### Screening

Title and abstract screening were conducted by two authors independently, with conflicts resolved through consensus or consultation with a more senior author if no consensus was reached. During the full‐text screening stage, studies were independently screened by the initial two authors, and disagreements were resolved in a similar manner.

### Assessment of agreement

Inter‐reviewer agreement was evaluated using the *κ*‐statistic for screening. Agreement was defined as follows: *κ* of 0.91–0.99 was considered to be almost perfect agreement; *κ* of 0.71–0.90 was considered to be considerable agreement; *κ* of 0.61–0.70 was considered to be high agreement; *κ* of 0.41–0.60 was considered to be moderate agreement; *κ* of 0.21–0.40 was considered to be fair agreement and a *κ* value of 0.20 or less was considered to be no agreement [22].

### Quality assessment

The methodological quality of non‐randomized studies was evaluated using the methodological index for non‐randomized studies (MINORS) criteria [41]. Using the items on the MINORS checklist, non‐comparative studies can achieve a maximum score of 16, while comparative studies can achieve a maximum score of 24. Non‐comparative studies were categorized a priori as follows: 0–4 indicates very low‐quality evidence, 5–7 indicates low quality, 8–12 indicates fair quality and scores ≥13 indicate high quality. For comparative studies, categorization was determined a priori as follows: 0–6 very low quality, 7–10 low quality, 11–15 fair quality, 16–20 good quality and ≥20 high quality [5]. Quality assessment of randomized controlled trials (RCTs) was conducted using the Cochrane risk‐of‐bias tool for randomized trials (RoB2) tool [15]. Five domains are evaluated: (1) bias from the randomization process, (2) bias due to deviations from intended interventions, (3) bias due to missing outcome data, (4) bias in the measurement of the outcome and (5) bias in the selection of the reported result. Each domain is categorized as either (1) low risk of bias, (2) some concerns or (3) high risk of bias. The overall risk of bias was considered to be ‘low risk’ for studies assessed to be at low risk of bias in all domains, ‘some concerns’ if the study raised some concerns in at least one domain and ‘high risk’ if the study was judged to be a high risk of bias in at least one domain, or if the study raised some concerns in multiple domains [15]. The quality assessment was performed in duplicate and the average calculated score was reported.

### Data abstraction

Data were extracted in an electronic spreadsheet designed a priori (Google Sheets; Google LLC). Extracted data included study characteristics (e.g., author(s), year of publication and level of evidence) and demographic data (e.g., number of patients, patient age and sex), follow‐up time and number lost to follow‐up. Knee details were also recorded (e.g., presence of loose bodies, trochlear dysplasia and Insall–Salvati score). The type of treatment given (surgical vs. conservative) was also included in the extraction.

### Outcome reporting and statistics

The primary outcome was the redislocation rate in paediatric patients undergoing surgical treatment compared to conservative treatment for acute, first‐time patellar dislocations. Secondary outcomes included functional and patient‐reported outcomes, such as Kujala scores and complications. Four meta‐analyses assessing the post‐operative pooled mean redislocation rate, subgroup mean redislocation rate in the included RCTs, subgroup mean redislocation rate in the two recent RCTs and a pooled mean post‐operative Kujala score comparing the two groups were completed. The meta‐analyses were performed using a random effects model (DataParty, Python 3.8.10). The Kujala Anterior Knee Pain Scale (AKPS) is a 13‐item patient‐reported assessment designed to assess patellofemoral pain in adolescents and young adults. The Kujala questionnaire is scored out of 100 points, with a lower score indicative of more subjective symptoms and functional limitations [20]. Forest plots were created to pool studies across the same outcome measures (DataParty, Python 3.8.10). The *I* ^2^ test was used to assess heterogeneity. Values of *I* ^2^ between 25% and 49% were considered ‘low’, 50%–74% ‘moderate’ and values greater than 75% were considered to be high statistical heterogeneity [16]. If the heterogeneity was considered ‘high’, the data would not be pooled together. Complications, aside from the redislocation rate, were classified using the Clavien–Dindo classification system [4].

## RESULTS

### Literature search

**Figure 1:**
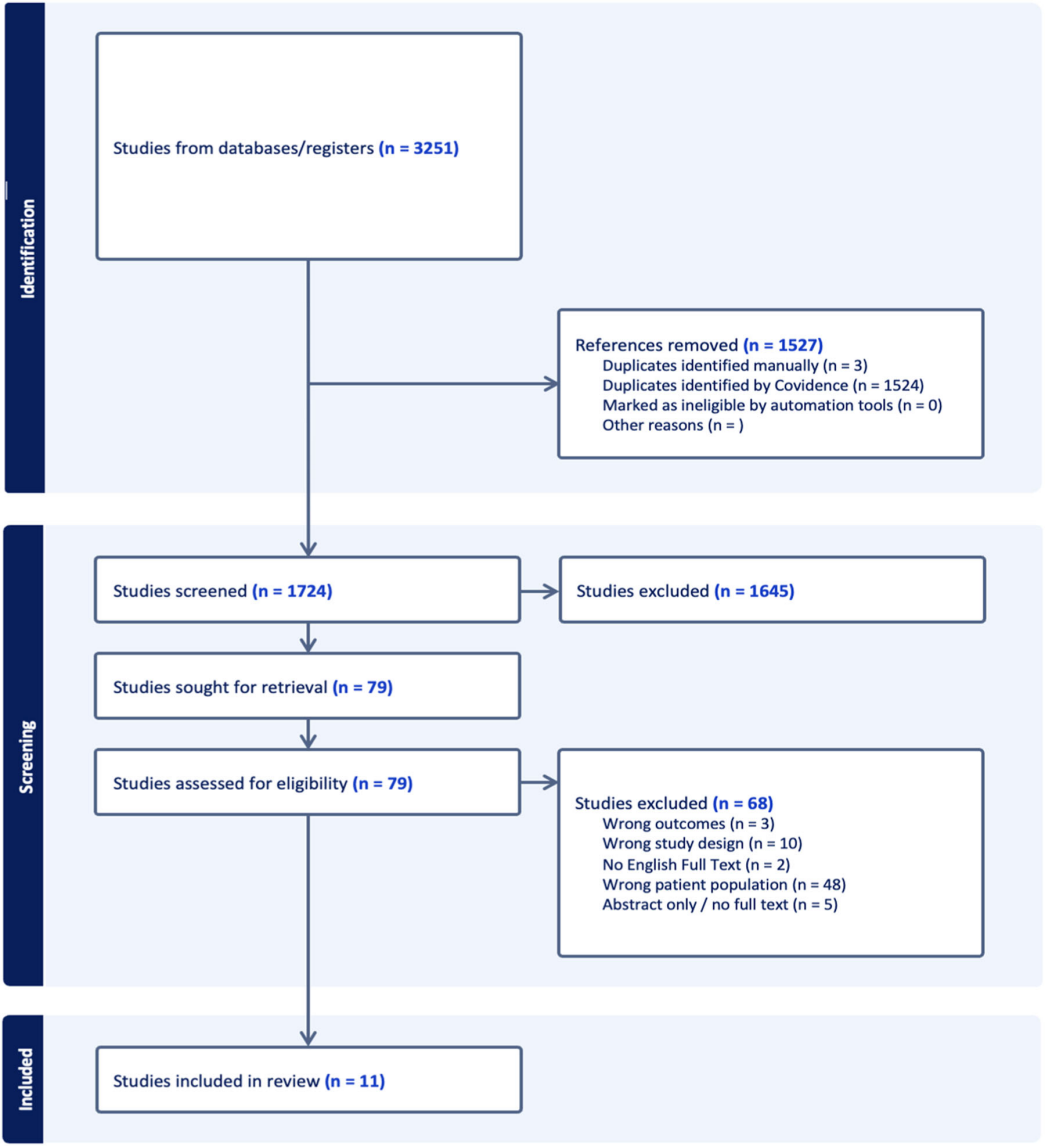
Preferred reporting items for systematic reviews and meta‐analyses flow diagram representing a systematic review on surgical versus conservative treatment of first‐time patellar dislocation in paediatric patients.

The initial literature search yielded 3251 studies, of which 1527 duplicates were removed. Among the remaining 1724 unique articles, 1645 were removed following title and abstract screening. Systematic screening and assessment of eligibility yielded 11 full‐text studies that satisfied inclusion criteria (Figure 1). Inter‐rater reliability analysis showed that substantial agreement and almost perfect agreement were achieved at the title and abstract screening stage (*k*, 0.70; 95% confidence interval [CI]: 0.62–0.79) and full‐text (*k*, 0.86; 95% CI: 0.70–1.0) stages of screening, respectively.

### Study quality

**Figure 2:**
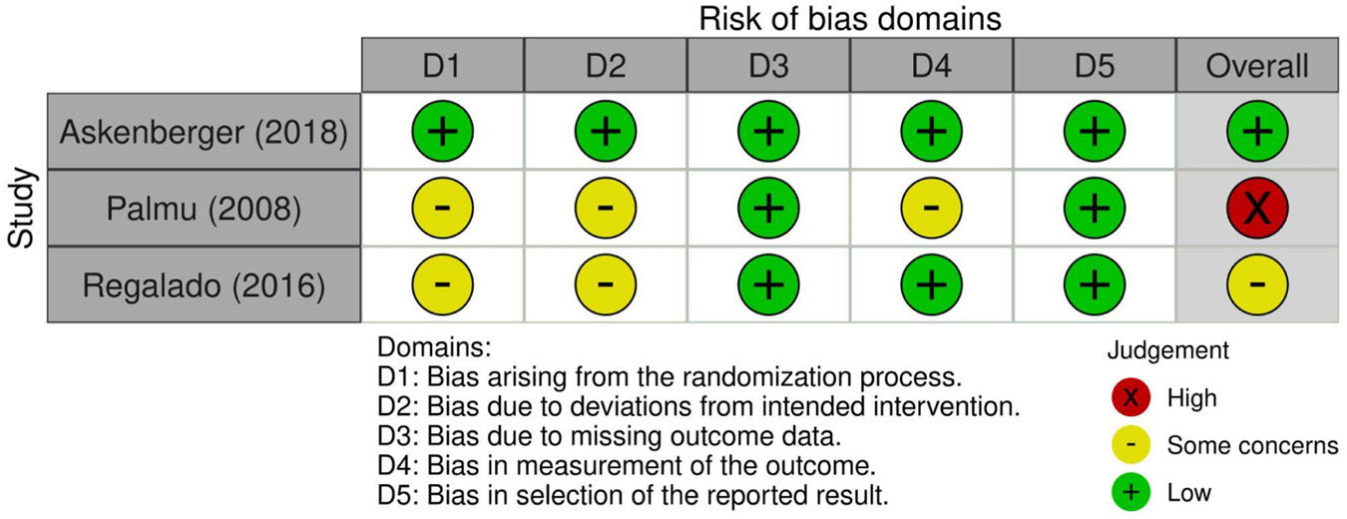
Traffic light plot demonstrating the risk of bias domains for the three randomized controlled trials that reported redislocation rates in a systematic review on surgical versus conservative treatment of first‐time patellar dislocation in paediatric patients.

Among the 11 studies included in this review, 4 (36.4%) were Level IV evidence [12, 13, 34, 38], 3 (27.3%) were Level III evidence [25, 30, 36], 3 (27.3%) were Level II evidence, including two of the three RCTs [1, 33, 35], and 1 (9.1%) was Level I evidence [2]. The mean MINORS score (Table 1) was 18.5 (77.1%) for comparative studies and 9.55 (59.7%) for non‐comparative studies. The Cochrane RoB‐2 scores were low risk of bias [2], high risk of bias [33] and some concerns for bias [35] among the three RCTs. The risk of bias for individual domains can be seen in Figure 2 [15].

**Table 1:** Study characteristics and outcomes.

Author (year)	Level of evidence	Mean MINORS score	Treatment group	Sample size	Mean follow‐up time (months)	Lost to follow‐up (%)	Female (%)	Mean age (years)	Redislocation rate (%)	Mean Kujala score
Apostolovic (2011)	II	72.9	Surgical	14	73.2	0	64.3	13.07	28.6	NR
			Conservative	23			82.6	14.3	17.4	
Askenberger (2018)	I	N/A	Surgical	37	24	0	49	13.2	21.6	90.9
			Conservative	37			54	13.03	43.2	95.9
Gurusamy (2021)	IV	75.0%	Surgical	30	31.2	19	43	14.2	10	92.7
Hartmann (2014)	IV	69.9	Surgical	13	110.7	0	46.2	14.7	0	87.2
Lewallen (2013)	III	71.9	Surgical	24	37.2	5	45.9	14.9	33.3	NR
			Conservative	198					38.4	
Mostrom (2014)	III	83.3	Operated during the acute phase	7	90	0	57.1	12.6	42.9	84
			Conservative	33			48.5	13.5	66.7	84
Palmu (2008)	II	N/A	Surgical	36	72	6	75	13	66.7	83
			Conservative	28			68	13	71.4	84
Pedowitz (2019)	IV	65.6	Surgical	41	49.2	21.1	46	13.8	61	No recurrent instability: 93.9 Recurrent instability: 83
Regalado (2016)	II	N/A	Surgical	16	72	6.7	68.8	13.5	31.2	NR
			Conservative	20		25	55	13.5	55	
Rueth (2023)	III	62.5	Surgical	101	32	10.9	49.5	14.8	0.9	85.3
Seeley (2013)	IV	53.1	Surgical (MPFL repair)	15	11.6	NR	30.4	14.6	20	NR

Abbreviations: MINORS, methodological index for non‐randomized studies; MPFL, medial patellofemoral ligament.

### Study characteristics

This study included 673 paediatric patients who underwent surgical or conservative treatment for acute, first‐time patellar dislocation (Table 1). Overall, 334 patients underwent surgical management, whereas 339 patients were managed conservatively. Four studies only evaluated patients undergoing surgical treatment [13, 36, 38]. Included patients had a weighted mean age of 13.9 (standard deviation [SD]: 0.72) years in the surgical groups, reported in nine studies [1, 2, 12, 13, 30, 33, 34, 35, 36], and a weighted mean age of 13.4 (SD: 0.51) years in the conservative groups, reported in five studies [1, 2, 12, 30, 33, 35]. Two studies reported overall mean age across both groups, with a weighted mean of 14.9 (SD: 0.21) years [25, 38]. The overall proportion of female patients was 57.1% (SD: 12.75) in the surgical group, reported in nine studies [1, 2, 12, 13, 30, 33, 34, 36], compared to 61.6% (SD: 13.74%) in the conservative group, reported in five studies [1, 2, 12, 30, 33, 35]. The mean follow‐up time across all included studies was 53.2 months (SD: 30.3 months, range 12–168 months) [1, 2, 12, 13, 25, 30, 33, 34, 35, 36, 38]. Among the 10 papers that reported the percentage of patients lost to follow‐up, the mean percentage was 8.0% (SD: 8.47%, range 0%–1.1%) [1, 2, 12, 13, 25, 30, 33, 34, 35, 36]. The one study that compared the percentage of patients lost to follow‐up between the surgical and conservative groups, rather than reporting a combined rate, reported values of 6.7% and 25%, respectively [35].

### Incidence of redislocation

**Figure 3:**
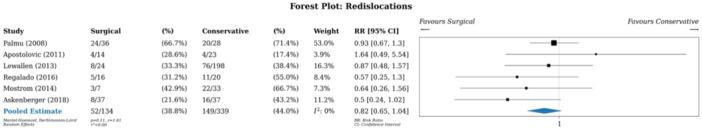
Forest plot (random effects) showing the overall pooled rates of redislocation across the surgical groups compared to the conservative groups with accompanying risk ratio calculations and 95% confidence intervals.

**Figure 4:**
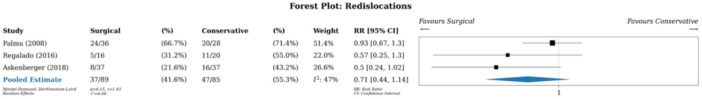
Forest plot (random effects) showing the overall combined rates of redislocation across the surgical groups compared to the conservative groups in the three included RCTs with accompanying risk ratio calculations and 95% confidence intervals. RCT, randomized controlled trial.

**Figure 5:**
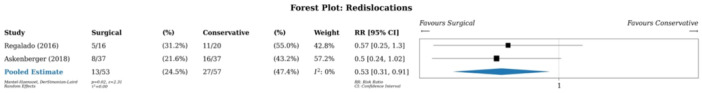
Forest plot (random effects) showing the overall rates of redislocation across the surgical groups compared to the conservative groups in the two recent RCTs, with accompanying risk ratio calculations and 95% confidence intervals. RCT, randomized controlled trial.

The weighted mean post‐operative rate of redislocation reported in eleven surgical groups comprising 334 patients was 25.1% [1, 2, 12, 13, 25, 30, 33, 34, 35, 36, 38], compared to a weighted mean of 44.6% reported in six studies across the conservative groups, comprising 339 patients [1, 2, 12, 25, 30, 33, 34, 35]. The meta‐analysis conducted to compare the rates of redislocation found in these six studies calculated a risk ratio (RR) of 0.82 (95% CI: 0.65–1.04, *I* ^2^ = 0%, *p* = 0.11), favouring the surgical treatment groups (Figure 3). The control group in one study was not included in the meta‐analysis as it included patients who underwent MPFL repair and non‐operative treatment in the same cohort [12]. A subgroup meta‐analysis of the rates of redislocation in the three RCTs comprising 174 patients demonstrated an RR of 0.71 (95% CI: 0.44–1.14, *I* ^2^ = 47%, *p* = 0.15), favouring the surgical group (Figure 4). In excluding an older RCT that did not stratify data based on the procedure and used outdated operative techniques, a subgroup meta‐analysis conducted on two recent RCTs comprising 110 patients demonstrated a statistically significant RR of redislocation of 0.53 (95% CI: 0.31–0.91, *I* ^2^ = 0%, *p* = 0.02) favouring the surgical group (Figure 5).

### MPFL reconstruction

Two studies examined MPFL reconstruction. One study comprising 101 patients with a mean follow‐up of 32.0 ± 12 found that only one patient (0.9%) suffered a recurrent dislocation after MPFLR [36]. Another study compared MPFLR to a control group of patients who underwent MPFL repair or non‐operative treatment and found that those who had an MPFLR had a 10% rate of redislocation, compared to 59% in the control group (*p* < 0.001) [12]. The weighted average of redislocation in patients undergoing MPFLR was 3.1%, compared to 39.4% in all other surgical procedures not involving MPFLR.

### Kujala score

**Figure 6:**
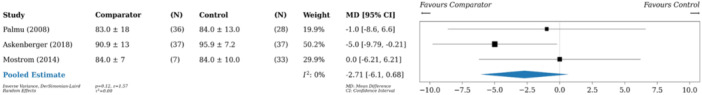
Forest plot (random effects) showing the overall pooled post‐operative Kujala score across the surgical groups compared to the conservative groups with accompanying mean difference values and 95% confidence intervals.

The weighted mean post‐operative Kujala score among patients in the surgical groups, reported in six studies comprising 224 patients, was 86.9 (SD: 3.9) [2, 12, 13, 30, 33, 36], compared to a mean score of 88.5 (SD: 6.9) in three studies among 98 patients in the conservative groups [2, 12, 30, 33]. One study found a significantly better Kujala score among patients who did not experience recurrent instability (mean [SD]: 93.9 [7.2]) than those who did (mean [SD]: 83.0 [11.7]) [34]. The findings of the meta‐analysis conducted for three 2‐arm studies comparing surgical to conservative treatment reported no statistically significant difference in mean Kujala score, with a mean difference of −2.7 (95% CI: −6.1 to 0.68, *I* ^2^ = 0%, *p* = 0.12), favouring the conservative group (Figure 6).

### Other complications

Five studies reported complications categorized as at least a Grade II at follow‐up [2, 12, 34, 35, 36]. Within the conservative treatment groups, comprising 126 patients, there were 0 recorded cases of complications categorized higher than Grade I. Across the operative treatment groups, comprising 239 patients, 7 (2.9%) patients were recorded to have complications [2, 12, 34, 35, 36] categorized as III‐b [7]. The complications included a second operation needed to remove implants (*n* = 6, 2.5%) [2, 12, 34, 36] and protrusion of the femoral screw requiring surgical revision (*n* = 1, 0.4%).

### Treatment protocols

Across the eleven studies, various operations and conservative management protocols are described. Surgical management involved MPFL repair [2, 12, 25, 33, 34, 38], lateral release [13, 25, 30, 33, 35], loose body removal [25, 34, 38], Roux‐Goldthwait procedure [30, 35], MPFL reconstruction [12, 36], arthroscopy [25, 38], medial retinacular and capsular repair with modified Yamamoto technique [1], mini‐open plication of the medial retinaculum [13], arthrotomy [38], open reduction internal fixation [25] and Roux‐Elmslie‐Trillat procedures [30]. Regarding conservative management plans, four studies reported the use of a knee brace and a physiotherapy regimen [2, 30, 33, 35], and two studies recorded a closed reduction of the dislocated patella, followed by knee immobilization in addition to strengthening exercises and/or a physiotherapy regimen [1, 25].

## DISCUSSION

The primary finding of this review is that early surgical treatment may reduce the risk of redislocation with no statistically significant effect on subjective outcomes or complication rates.

The management of patellar dislocation is critical in the paediatric population, as they are more likely to experience recurrence compared to their adult counterparts [19], which puts them at a higher risk of long‐term complications, such as knee pain, functional limitations and early arthritis [43]. Two previous systematic reviews and meta‐analyses looked at three and four studies, respectively, and concluded that early surgery did not significantly improve clinical outcomes compared to conservative management [10, 24]. These reviews highlight one study [35] that shows a statistically significant benefit towards surgery. However, the pooled effects of these analyses fail to support this finding. This may be because the included studies incorporated different surgical techniques in other patients, such as LRR, medial imbrication and MFPLR, without stratifying results, highlighting one of the main challenges of extrapolating results from these comparative trials. This current review aims to build off the previous works by including more studies, encapsulating the entire scope of the literature on surgical treatment for first‐time patellar dislocations in paediatrics.

The promise of early surgical intervention can be seen in studies that standardize surgical procedures. One study examined 101 patients who underwent isolated MPFLR and found that only one patient suffered a dislocation at a mean follow‐up of 32.0 ± 12.1 months [36]. Another study looked at a subgroup of patients who underwent mini‐open medial reefing and arthroscopic lateral release and found that none suffered recurrent dislocation at a mean of 110.7 months [13]. The three RCTs included in this review also provide valuable information as higher‐quality evidence. One study [2] examined isolated MPFL repair alone and showed lower dislocation and statistically significant improvement in Kujala. Another looked at LRR and a modified Roux‐Goldwait procedure, which resulted in zero dislocations in 3 years and five (33%) in 6 years, which was significantly better than the conservative group [35]. The modified Roux‐Goldwaith combines proximal and distal realignment with LRR and medial. This was one of only two studies that incorporated bony alignment procedures. This is likely because these are more complex and risky procedures in the paediatric population, as surgeons must be careful not to compromise the physis. The final RCT included patients treated with LRR, medial tissue repair (MTR) and a combination of both, but did not stratify results based on procedure [33] and did not show statistically significant improvements with surgery. This study, performed in 2008, also acknowledges that the surgical procedures used were not current for the time [31, 33], as the MPFL was repaired via direct suturing in a dysplastic patellofemoral joint, as opposed to more modern repairs with suture anchoring [17].

The higher dislocation rates seen in the conservative groups may be due to the inconsistent rehabilitation protocols, with discrepancies in brace use, icing and physiotherapy regimens. Even with extensive physiotherapy, a dislocation and subsequent tear of the MPFL may outweigh medial strength improvements, as the MPFL acts as the primary restraint to lateral translation of the patella between 0‐30 degrees of flexion [27]. In adults, primary reconstruction and repair of the MPFL results in lower redislocation rates and improved Kujala scores compared to rehabilitation alone [5, 23]. Some authors argue that although an isolated MPFL reconstruction is an option for first‐time dislocations of a growing child, it is rarely justified [40]. However, the results described in this review suggest that reconstructing the MPFL can result in better overall outcomes than non‐operative means. Currently, there remains insufficient evidence for a complete shift towards early operative treatment for first‐time patellar dislocations in paediatric populations; however, it is necessary for further research to focus on the potential of MPFLR, which was the most beneficial operation in this review.

The strength of this systematic review is that it is a comprehensive analysis of all available studies on surgical management of first‐time patellar dislocations in a paediatric population. This study employed rigorous methodology and utilized multiple meta‐analyses to determine redislocation rates, along with Kujala scores. The limitations of this paper stem from the quality of evidence available addressing this research question. Only three RCTs were included, and over half the included studies were of Level III or IV evidence, which is why a separate subgroup meta‐analysis was performed on the RCTs of Levels I and II evidence. Another limitation is the inconsistency and discrepancy in the discussed surgical techniques. Many included studies incorporated multiple surgical techniques in their patient population without presenting their results separately. Furthermore, there was notable variation in conservative protocol between studies. The inconsistent and potentially suboptimal regimens of conservative protocols included may have introduced bias favouring surgery. Ultimately, these factors make it difficult to determine which, if any, of the proposed surgical techniques are truly superior or inferior to conservative modalities.

Moreover, while this paper only included patients under 18, some studies included patients with closed physis without stratifying results, while others did not comment on physeal status. This provides an additional challenge, as physeal status plays a significant role in the surgical treatment and outcomes after patellar dislocations [44]. Finally, predictive factors for redislocation were not analysed or stratified in this analysis, as they were presented in an inconsistent fashion among the included studies. This limitation may have contributed to bias in our findings. Ultimately, individualized treatment plans should be made to account for the root cause of instability when present. Future studies should focus on comparing MPFLR to conservative measures to better understand how to preserve bony health and cartilage in young patients with patellar dislocations.

## CONCLUSION

This review demonstrated that early surgical intervention for first‐time patellar dislocations in a paediatric population may lower redislocation rates without affecting subjective outcomes. This reduction is most notable in procedures that involve the reconstruction of the MPFL. Due to the inconsistency of protocols used in both groups, it is difficult to denote a definitive benefit of surgery. Individual patient factors, such as anatomical abnormalities and physeal status, should be considered when creating a management plan for these patients.

## AUTHOR CONTRIBUTIONS

All co‐authors participated in the development of this research paper. Benjamin Blackman and Joshua Dworsky‐Fried developed the search strategy, and performed article screening, data extraction, quality assessment, data analysis and manuscript writing. Dan Cohen assisted with the search strategy, data analysis and manuscript writing. Benjamin Blackman and Joshua Dworsky‐Fried prepared tables and figures. Benjamin Blackman, Joshua Dworsky‐Fried, Dan Cohen, David Slawaska‐Eng, Lauren Gyemi, Nicole Simunovicc, Devin Peterson, Olufemi R. Ayeni and Darren de SA reviewed and revised further versions of the manuscript and approved the final version.

## CONFLICT OF INTEREST STATEMENT

The authors declare no conflict of interest.

## ETHICS STATEMENT

This study does not contain any studies with human participants or animals performed by any of the authors.

## Supporting information

Appendix S1

## Data Availability

Data can be made available upon reasonable request at bwb531@gmail.com.
