# Supplementary figures and images for: Surgical management of first‐time patellar dislocations in paediatric patients may lower rates of redislocation compared to conservative management: A systematic review and meta‐analysis
Source: Knee Surg Sports Traumatol Arthrosc. 2024 Oct 30;33(6):2156–66. doi: 10.1002/ksa.12524 (PMC12104781; doi:10.1002/ksa.12524)

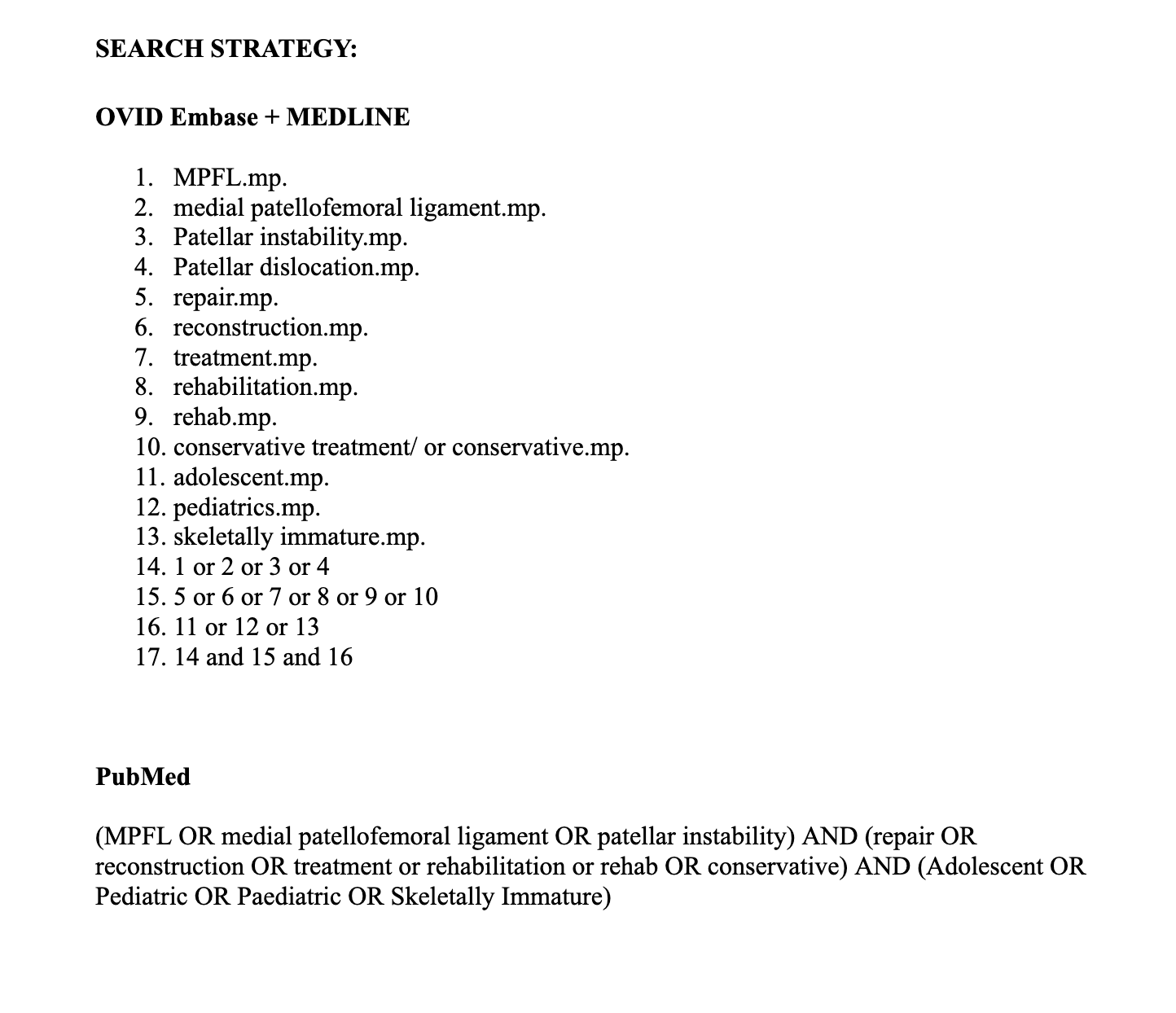

Supplement: Supplementary file 1 — Appendix S1 [file KSA-33-2156-s001.png]
